# Effects of exercise training and photobiomodulation therapy (EXTRAPHOTO) on pain in women with fibromyalgia and temporomandibular disorder: study protocol for a randomized controlled trial

**DOI:** 10.1186/s13063-015-0765-3

**Published:** 2015-06-04

**Authors:** Mariana Moreira da Silva, Regiane Albertini, Ernesto Cesar Pinto Leal-Junior, Paulo de Tarso Camillo de Carvalho, José Antonio Silva, Sandra Kalil Bussadori, Luis Vicente Franco de Oliveira, Cezar Augusto Souza Casarin, Erinaldo Luiz Andrade, Danilo Sales Bocalini, Andrey Jorge Serra

**Affiliations:** Nove de Julho University, Rua Vergueiro, 235, Liberdade, São Paulo, SP 01504-000 Brazil

**Keywords:** Exercise training, Fibromyalgia, Light-emitting diode, Low-level laser therapy, Pain, Phototherapy, Temporomandibular joint disorder

## Abstract

**Background:**

Fibromyalgia (FM) is a syndrome most prevalent in women, in whom it is characterized mainly by chronic pain. An important issue is that many patients with FM are reported to have temporomandibular dysfunction (TMD), and the coexistence of these pathologies generates a clinical outcome of high complexity. The literature is unclear regarding an effective therapy for reducing pain in patients with both comorbidities. Exercise training and phototherapy (low-level laser therapy with light-emitting diode) are two of the approaches used to treat pain. Thus, the aim of this study is to assess the potential role of exercise training plus phototherapy in reducing chronic pain in women with FM and TMD. A further aim is to determine whether the interventions can improve quality of life and modulate endogenous serotonin.

**Methods/Design:**

A randomized controlled clinical trial will be conducted. It will involve 60 women ≥ 35 years of age with a diagnosis of FM and TMD. After recruitment, patients will be randomly allocated to one of four groups: a control group (no intervention), a group that will receive a phototherapy intervention (PHO), a group that will be prescribed muscle-stretching, aerobic, and facial exercises (EXT), or a group that will receive phototherapy plus exercise interventions (PHO + EXT). The trial will last 10 weeks, and the following outcomes will be evaluated on two separate occasions (baseline and within 24 h after the last day of the protocol). Pain intensity will be analyzed using a visual analogue scale and the McGill Pain Questionnaire, and pain thresholds will be punctuated using a digital algometer. FM symptoms will be assessed using the Fibromyalgia Impact Questionnaire, and quality of life will be determined with the 36-item Short Form Health Survey. Serotonin levels will be evaluated in salivary samples using a competitive enzyme-linked immunosorbent assay.

**Discussion:**

This is the first randomized controlled trial in which the role of phototherapy, exercise training, and a combination of these interventions will be evaluated for chronic pain in patients with FM and TMD. The results will offer valuable clinical evidence for objective assessment of the potential benefits and risks of procedures.

**Trial registration:**

ClinicalTrials.gov Identifier: NCT02279225. Registered 27 October 2014.

## Background

Fibromyalgia (FM) is a chronic syndrome characterized mainly by pain, nonrestorative sleep, fatigue, morning stiffness, depression, and cognitive disorders [1, 2]. Patients with FM often show widespread pain in the presence of tender points (expressed as mild or greater tenderness), which provide the most sensitive, specific, and accurate criteria for making the diagnosis [1, 3]. Using this classification, almost all patients with FM are women because they have more tender points than do men [4]. Overall, FM symptoms lead to significant reduction in functional capacity and quality of life [2, 5, 6]. Developing treatment teams is useful, including clinicians with expertise in patient education, exercise training interventions, and cognitive behavioral therapy [7–9]. An optimized treatment can be provided using the following steps: (1) overall recommendation (e.g., patient education), (2) nonpharmacologic (e.g., exercise, cognitive behavioral therapy, alternative medicine, and central nervous system neurostimulatory intervention), and (3) pharmacologic (e.g., tricyclic complexes, gabapentinoids, γ-hydroxybutyrate, naltrexone, cannabinoids, selective serotonin reuptake antagonists, nonsteroidal anti-inflammatory drugs, and opioids) [4].

A key issue is the association of FM with other musculoskeletal comorbidities, such as temporomandibular dysfunction (TMD) [5]. It has been shown that TMD can develop as a result of mandibular compression during daily living activities and sleep in the patients with FM [5–7], in which the coexistence of these pathologies generates a clinical outcome of high complexity [8]. Unfortunately, no published studies of patients with FM and TMD have evaluated an effective pharmacologic and/or nonpharmacologic therapeutic intervention. Previous studies have examined only the role of different interventions in the situation of a pathology per se. In this issue, exercise training is highly recommended in the controlling of patients with FM. Aerobic, strength, and mixed training programs (combination of aerobic, strength, and flexibility) were shown to reduce pain, number of tender points, fatigue, depression, and anxiety and to improve health-related quality of life as well as functional capacity [9, 10]. Exercises targeted for the face are also indicated for treatment of TMD as a procedure combined with other therapies (e.g., electrotherapy, physiotherapy, temporomandibular joint mobilization, and facial massage) to improve pain sensitivity [11–13].

Phototherapy, such as low-level laser therapy (LLLT) and light-emitting diode (LED) therapy, are recently developed options to treat FM and TMD [14]. LLLT is assumed to modulate several signaling pathways and physiologic mechanisms involved in analgesia [15–17]. This therapeutic approach seems to increase β-endorphin levels, lymphatic flow, and blood supply. Moreover, LLLT is reported to reduce bradykinin, histamine release, swelling, pain-associated molecules, and inflammation phase and to induce muscle relaxation [18, 19]. Similar results have been shown with LED therapy, which has a minor cost and better equipment durability [20].

A comprehensive review of the literature revealed no studies evaluating the role of phototherapy with multiple light sources (LLLT and LED) on the same device in the pain condition in patients with FM and TMD. In addition, there are no prior data involving a combined intervention of phototherapy and exercise training in patients with FM and TMD. The hypothesis of the proposed study is that combined intervention is capable of improving chronic pain in patients with FM and TMD. Knowledge regarding the clinical effects of these interventions can contribute to improved rehabilitation and quality of life among such patients.

### Objective

In this trial, we seek to assess the potential role of exercise training plus phototherapy to reduce chronic pain in women with FM and TMD.

## Methods/Design

### Study design

**Fig. 1:**
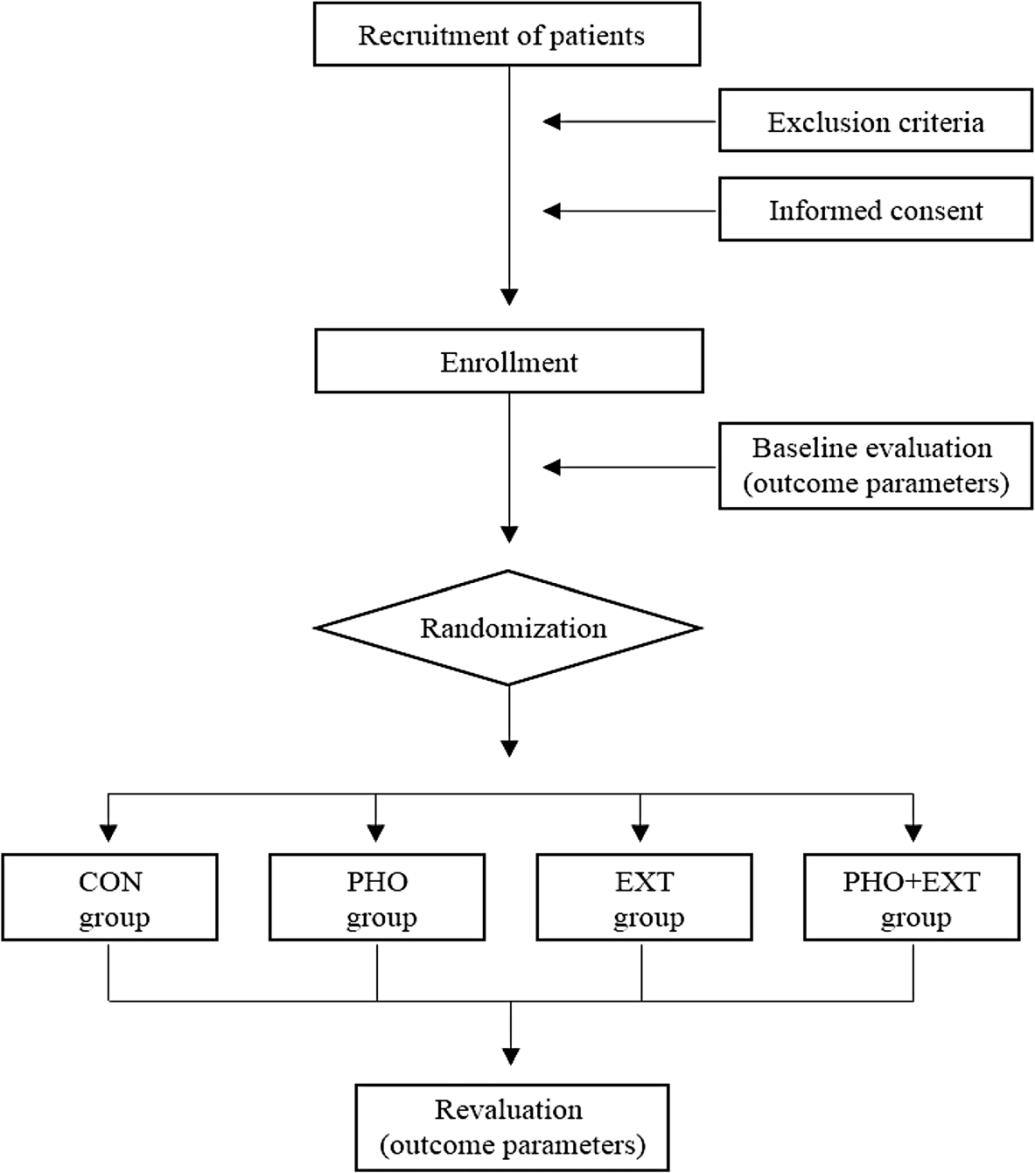
Trial scheme. CON, Control; EXT, Exercise training; PHO + EXT, Phototherapy plus exercise training; PHO, Phototherapy

The EXTRAPHOTO trial (ClinicalTrials.gov identifier NCT02279225) is a multicenter randomized controlled clinical trial comparing the use of exercise training, phototherapy, and exercise training plus phototherapy for pain control in women with two comorbidities (FM and TMD). As part of a simultaneous recruitment process, in the EXTRAPHOTO trial we will identify participants with target disorders in a database of the Unified Health System of Sao Paulo, Brazil. A preliminary analysis showed that patients at three centers with FM specialties are eligible: (1) the Rheumatology Clinic of Nove de Julho University, Sao Paulo, Brazil; (2) the basic health center of Sao Paulo, Brazil; and (3) the basic health center of Sao Paulo in Sao Bernardo do Campo, Brazil. The study procedure will follow the guidelines of the Consolidated Standards of Reporting Trials statement. All participants will receive data on the study aims and experimental procedures. We will obtain signed, written informed consent forms from each patient before inclusion in the study. The trial scheme is detailed in Fig. 1.

### Population and sample size

In a literature review, we found that FM prevalence is higher in women between ages 35 and 60 years than in men [21]. All women patients aged ≥ 35 years and referred for FM and TMD diagnostic evaluation were eligible for inclusion in the present trial. Patient eligibility comprises an evaluation of medical history, physical examination, and dental and rheumatologic routine evaluations. Moreover, patients had to have undergone FM screening as reported by the American College of Rheumatology using the Fibromyalgia Impact Questionnaire (FIQ) 1. The TMD diagnosis had to be reported on the basis of TMD research diagnostic criteria (axes I and II) [22]. The sample size calculation was based on a previous study by Consalter et al. [23] in which the TMD prevalence was 80 % in patients with FM. A 95 % reliability and a 10 % error rate was assumed to estimate the sample size (n) as follows [24]:

$$ n={\left(\frac{z_{a/2}\sqrt{p\left(1-p\right)}}{\varepsilon}\right)}^2={\left(\frac{1.96\ast \sqrt{0.8\ast 0.2}}{0.1}\right)}^2 $$

A total of 60 women will be randomized for this trial, with 20 women allocated per group.

### Recruitment and inclusion criteria

The recruitment methods include direct referral of patients who consult for FM, in which TMD also will be investigated. A rheumatologist and a dentist will make the diagnoses of FM and TMD, respectively. A physician at each FM medical center will participate. A compatible diagnosis of FM will be obtained on the basis of evaluation criteria similar to those previously well defined [25]. The recruitment of 60 patients is planned to be finished within 24 months. Inclusion criteria are as follows: women ≥ 35 years of age presenting with at least a 5-year diagnosis of FM and TMD, optimized drug treatment, cognitive independence to respond to inquiries, functionally independent regarding daily physical activity, availability and ability to fully comply with the training process and phototherapy, and no contraindication to the research procedures. All centers will be informed about the EXTRAPHOTO trial and its inclusion criteria during department meetings. Exclusion criteria are patients in a prior regular and structured physical activity program; missing more than three times from treatment; presence of psychiatric disorders, missing teeth, and/or use of dentures; a history of trauma to the face; currently undergoing orthodontic intervention; and any contraindication to exercise or phototherapy. The FM symptoms can be confused with other disorders. Thus, women for whom there are suspicions of any of the following disorders will be excluded: osteoarthritis, bursitis, tendinitis, rheumatoid arthritis, palindromic rheumatism, polymyalgic rheumatic disease, hydroxyapatite crystal diseases, systemic lupus erythematous, dermatomyositis-polymyositis complex, Lyme disease, hypothyroidism or hyperthyroidism, hyperparathyroidism, previous history of hepatitis, and history for Epstein-Barr virus infection. Sjögren, McArdle, Addison, Cushing, and paraneoplastic syndromes will also be exclusion criteria.

### Randomization, blinding, and experimental groups

The EXTRAPHOTO trialists will enroll patients immediately after diagnosis of the two comorbidities and referral from recruitment centers. Patient enrollment and randomization, as well as data management, will be carried out by the Program in Biophotonics Applied to Health Sciences of the Nove de Julho University. The blinding will be applied to patients receiving phototherapy and a researcher reported to guide the exercise training. Thus, a researcher will be responsible for programming the phototherapy device, which can be turned on (phototherapy) or off (placebo) prior to application based on the randomization. A second researcher will guide the exercise training and will be blinded for phototherapy and/or placebo procedures. A third investigator will be blinded to allocation and will independently assess the outcomes. The statistical analysis will be performed by a fourth researcher, who will also be blinded to allocation of the patients.

The eligible participants will be instructed not to change their lifestyle or pharmacologic therapy during the study and will be randomized to one of the following groups:

1. Control (CON): patients not subjected to any intervention; the phototherapy device will be turned off (placebo) as a blinded procedure for these participants
2. Phototherapy (PHO): patients subjected to phototherapy
3. Exercise training (EXT): patients subjected to aerobic exercise; the phototherapy device will be turned off (placebo) as a blinded procedure for these participants
4. Phototherapy plus exercise training (PHO + EXT): patients subjected to phototherapy and aerobic exercise training

### Interventions

The trial will be 10 weeks long, during which time patients will undergo two sets of phototherapy, exercise, or placebo procedures per week. Phototherapy will be applied 30 min before each exercise bout, and treatment sessions will be carried out Tuesdays and Thursdays each week. For eligible patients, the research will record outcome parameters at baseline prior randomization and 48 h after the last day of intervention. This evaluator will be blinded to the allocation of the patients into the respective groups.

### Assessment and result reliability

Two therapists will guide the interventions at each basic health center. To ensure the feasibility of the study and the reliability of the results, all therapists and researchers will be trained in data collection procedures before the start of the trial. Moreover, the results will be analyzed by an independent investigator without knowledge of the allocation of patients to their experimental groups.

### Phototherapy

**Fig. 2:**
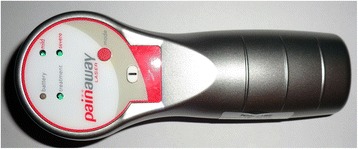
Phototherapy device that will be used to treat pain

Phototherapy will be carried out with a PainAway™ nine-diode cluster portable device (Multi Radiance Medical, Solon, OH, USA). This portable device was specifically designed for pain relief and has two operating modes: mild and severe. Because of the characteristics of pain that patients with FM, we decided to use the device at the mild setting. The device has two identical application hand pieces—one active tip and the other for placebo procedure without energy, both equipped with a similar sound device. These equipment pieces are required to blinding applicator research and patients. Thus, the researcher who will apply phototherapy and the patient will not know which tip was used (active or placebo). The irradiation will be applied only in the tender points in which pain has been reported by the participants. These tender points can be occipital, cervical (near the C7), trapezius, supraspinatus, second costochondral joint, lateral epicondyle and gluteal/sacrum, and greater trochanter on the medial knee border. The temporomandibular joint (bilaterally) will be another irradiation target because of TMD. Each point will be irradiated for 300 s, in which 39.3 J of total energy will be delivered for each irradiation point. An independent researcher will be responsible for controlling the on or off phototherapy device because therapists and patients will be blinded to the procedure. The applicator researcher and patient will be wearing eye protection devices. The CON group will be subjected to the same procedures as the groups subjected to the PHO intervention. The phototherapy device to be used is shown in Fig. 2. All parameters of the phototherapy device are shown in the Table 1.

**Table 1:** Phototherapy parameters

Parameters	Pulse
	1 Superpulsed infrared
Wavelength of lasers (nm)	905
Frequency (Hz)	1000
Average optical output (mW)	0.9
Power density (mW/cm^2^)	2.25
Peak power (W)	8.5
Dose (J)	0.3
Energy density (J/cm^2^)	0.75
Spot size of laser (cm^2^)	0.4
Number of LEDs	4 Red
Wavelength of LEDs (nm)	640 (±10)
Frequency (Hz)	2
Average optical output (mW), each	15
Power density (mW/cm^2^), each	16.66
Dose (J), each	4.5
Energy density (J/cm^2^), each	5
Spot size of LED (cm^2^), each	0.9
Number of LEDs	4 Infrared
Wavelength of LEDs (nm)	875 (±10)
Frequency (Hz)	16
Average optical output (mW), each	17.5
Power density (mW/cm^2^), each	19.44
Dose (J), each	5.25
Energy density (J/cm^2^), each	5.83
Spot size of LED (cm^2^), each	0.9
Magnetic field (mT)	35
Treatment time (s)	300
Aperture of device (cm^2^)	4
Total energy delivered (J)	39.3

*LED* light-emitting diode

### Exercise training protocol

The EXT protocol will be carried out according to the guidelines of the American College of Sports Medicine [26], and a previously published protocol will be applied for the treatment of tender points of the patients with FM [25]. The protocol consists of stretching and aerobic exercise two times per week for 10 weeks. Active static stretching will be applied to the following muscle groups: biceps, trapezius, latissimus dorsi, pectoralis, paraspinal, hamstrings, and quadriceps. Each stretching exercise will be performed three times for 30 s, and 30 s of rest will be allowed between each stretch. The stretch will be conducted to produce mild discomfort. The aerobic exercise will be carried out on an electronic motorized drive (LX-150; Movement, Sao Paulo, Brazil) without inclination for 30 min per session. The exercise will be guided at an intensity of 75 % of age-predicted maximum heart rate (220 − age in years).

Exercises for TMD will be carried out as reported in detail elsewhere [27]. Exercises will be performed with the patient in a sitting position and repeated three times for every movement. A maximum oral opening will be required for the first exercise; the second exercise will be a tongue slippage on the palate; and the third exercise will be oral lateralization to the right and left with contraction of the masseter muscle. This exercise will be conducted with the participant’s mouth filled with air for 3 s. Ultimately, circular fingertip motions will be applied with slight pressure on the temporomandibular joint and masseter muscle [27, 28].

### Outcome parameters

The outcome parameters described below will be evaluated at baseline and 24 h after the last day of the experimental protocol.

#### Personal and anthropometric data

We will collect data on participants’ occupation, education level, marital status, social security number, home address, age, body weight, and height.

#### Overall parameters of pain

To assess overall parameters of pain, we will gather information on daily duration of pain, pain tender points, number of days with a high pain level, sleep quality, main features that show accentuation or worsening of pain, incidence of stiffness and pain, jaw locking, and morning fatigue.

#### Visual analogue scale

We will use a visual analogue scale (VAS) to assess pain level. The VAS consists of a limited strip of 10 cm in length. This strip has at its ends the anchor terms *no pain* and *worst pain possible*. The patient will be instructed to report the level of pain sensation to a point along this line, with the scores ranging from 0 to 10 and obtained by measuring the distance between the end anchored by the term *without pain* and the point marked by the participant [29]. Additional analysis will include changes from randomization to the end of withdrawal (week 10) in an adapted Portuguese version of the McGill Pain Questionnaire (McPQ) [30].

#### Algometry

A digital algometer (DD-200; Instrutherm, Sao Paulo, Brazil) will be used to evaluate pain sensibility through the application of pressure. The algometer will be positioned to read specific FM tender points and TMD joints using the rubber tip measuring 1 cm^2^ in direct contact with the skin. A gradually increasing pressure will be applied to all points until the patient reports feeling pain, at which point the researcher will stop applying pressure and display values will be recorded. The procedures will be performed only once to each point, and a 30-s rest period will be given between readings [29].

#### Fibromyalgia symptoms and quality of life

Outcome parameters will be evaluated with the FIQ. The FIQ is a multidimensional instrument developed to assess several FM symptoms (pain, fatigue, stiffness, tenderness, sleep disorders, depressed mood, anxiousness, problems with memory, imbalance, sensitivity to non-painful stimuli, and performing daily tasks) [29]. The Medical Outcomes Study 36-item Short Form Health Survey (SF-36) will also be used [29].

#### Salivary serotonin

Concentration of serotonin in the saliva is relevant to nociception and the pathogenesis of chronic pain syndromes seen in FM [31]. Thus, mean serotonin changes from randomization to the end of withdrawal will be evaluated in 25-μl salivary samples by using a competitive enzyme-linked immunosorbent assay (ELISA) according to the manufacturer’s instructions (Human Serotonin ST ELISA Kit; MyBioSource, San Diego, CA, USA). The samples will be collected with cotton swabs into plastic tubes without any stimulation and frozen (−80 °C). Patients will be instructed to rinse their mouths with water and not to eat or drink 30 min before the samples are collected. The ELISA plates will be read with a SpectraMax Plus 384 spectrophotometer (Molecular Dynamics, Sunnyvale, CA, USA) at a wavelength of 400 nm [31].

### Statistical analysis

Pain will be the primary endpoint and will be determined using a VAS, an algometer, the FIQ, and the McPQ. Quality of life as assessed by SF-36, salivary serotonin level, and overall parameters of pain will be secondary endpoints. Statistical analysis will be performed using IBM SPSS software (version 13.0; IBM, Armonk, NY, USA), and a two-sided *P* value <0.05 will be considered significant. The intragroup and intergroup comparisons will be carried out with two-way repeated-measures analysis of variance with post hoc Bonferroni correction or the Kruskal-Wallis test with a post hoc Dunn test. The choice of tests will be based on the distribution (normal or non-normal) of the data by the Shapiro–Wilk test. For categorical data, χ^2^ statistics will be used.

### Ethical approval

The ethics committee of the Nove de Julho University (Sao Paulo, Brazil) reviewed and approved this study protocol (protocol number 419.828/2013). The trial was conceived and will be conducted according to the principles set forth in the Declaration of Helsinki [32]. All patients will be requested to provide written informed consent prior to randomization, using standard forms. Patients may withdraw from the study at any time without penalty. Our standpoint is that any intervention has beneficial effect. It is an ethical issue in which the control group knows the positive results of the study. Therefore, the control group will be given the opportunity to receive intervention after the end of the trial. The trial is registered at ClinicalTrials.gov under the number NCT02279225 (27 October 2014).

## Discussion

The findings this trial are predicted to offer evidence regarding the role of phototherapy and exercise training as well as a combined intervention in a multimodal management program for patients with FM and TMD. This is a target population because TMD prevalence is higher in patients with FM [28]. Pain is a the main symptom of these conditions [33–35], and it limits the patient’s activities of daily living, such as walking, carrying objects, occupational, eating, talking, yawning, and smiling, as well as a worsened in quality of life [35, 36]. Moreover, pain is associated to sleep disturbances, fatigue, and mood and memory disorders [36, 37]. Hence, we consider that pain may represent the main endpoint in the EXTRAPHOTO trial.

It has been proposed that chronic pain disorders are driven mainly by alterations in the central nervous system in which serotonin is considered the most important neurotransmitter that modulates endogenous mechanisms [38]. Because several studies have shown that serotoninergic dysfunction can mediate the pathophysiology of FM and TMD [38, 39], salivary serotonin level will represent a secondary endpoint in the EXTRAPHOTO trial. To the best of our knowledge, no previous randomized controlled trial has evaluated whether phototherapy and/or exercise training can modulate endogenous serotonin in patients with FM and TMD [40].

The EXTRAPHOTO trial is the first randomized controlled study evaluating the role of phototherapy, exercise training, and a combination of these interventions for chronic pain in patients with FM and TMD. In fact, the results of this trial will provide valuable clinical evidence for objective assessment of the potential benefits and risks of procedures. The results will be published after the trial is completed.

## Trial status

The proposed study is presently under development, and individuals are being allocated in the experimental groups.

## Abbreviations

CON: Control
ELISA: Enzyme-linked immunosorbent assay
EXT: Exercise training
FIQ: Fibromyalgia impact questionnaire
FM: Fibromyalgia
LED: Light-emitting diode
LLLT: Low-level laser therapy
PHO: Phototherapy
PHO + EXT: Phototherapy plus exercise training
TMD: Temporomandibular dysfunction
VAS: Visual analogue scale
